# Timely Revascularisation and Non-vitamin K Oral Anticoagulant (NOAC) Therapy Resulting in Resolution of Left Ventricular Apical Thrombus in a Young Female With Stumpless Left Anterior Descending Artery ST-Elevation Myocardial Infarction (LAD STEMI)

**DOI:** 10.7759/cureus.96453

**Published:** 2025-11-09

**Authors:** Ankit Gupta, Ojas Gowda S. G., Mahesh Mannava, Sakthivel Duraisamy, Sourav Ranjan Parija

**Affiliations:** 1 Cardiology, All India Institute of Medical Sciences, Raebareli, Raebareli, IND; 2 Internal Medicine, All India Institute of Medical Sciences, Raebareli, Raebareli, IND

**Keywords:** cardiac mri, coronary angiography, echocardiography, lv thrombus, noac therapy, stemi

## Abstract

Left ventricular (LV) thrombus is an uncommon but high-risk complication of acute anterior ST-elevation myocardial infarction (STEMI), particularly in young patients with large infarcts, which is an uncommon finding in the modern PCI era. Its management becomes more challenging when the culprit artery presents as a stumpless occlusion of the left anterior descending artery (LAD).

A 30-year-old female presented with angina (Class IIIB) and anterior wall STEMI. Echocardiography revealed a severely hypokinetic apical septal region with a large LV clot but preserved basal function and overall ejection fraction. Coronary angiography demonstrated a stumpless occlusion of the LAD. Percutaneous coronary intervention (PCI) was performed via radial route using a 6F Judkins Left guiding catheter (Cordis, USA). A Sion Blue wire (Asahi Intecc, Japan) was parked in the distal ramus for support; a Fielder XT wire (Asahi Intecc, Japan) over a Finecross microcatheter (Terumo, Japan) successfully crossed the lesion. Sequential pre-dilatation with Mini Trek balloons (Abbott Vascular, USA) was followed by deployment of a 3.0 × 24 mm Xience drug-eluting stent (Abbott Vascular, USA). The final angiogram showed Thrombolysis in Myocardial Infarction (TIMI) grade III flow. The patient was discharged on triple therapy (aspirin, clopidogrel, rivaroxaban). At two-month follow-up, echocardiography and cardiac MRI confirmed complete resolution of LV clot.

This case illustrates that revascularisation of the LAD not only restored perfusion but also improved regional wall motion, thereby reducing stasis and facilitating clot resolution when combined with anticoagulation. This synergistic effect highlights the importance of timely PCI and non-vitamin K oral anticoagulant (NOAC) therapy in preventing embolic complications in young STEMI patients with LV thrombus.

## Introduction

Left ventricular (LV) thrombus is a well-recognised complication of large anterior wall myocardial infarction, historically occurring in up to 30% of cases, but is now less common due to widespread use of primary percutaneous coronary intervention (PCI) [1,2]. LV thrombus carries a high risk of systemic embolisation, including stroke. While anticoagulation remains the cornerstone of management, the role of timely revascularisation in facilitating thrombus resolution is less recognised. One in four ischaemic strokes is cardioembolic (CE); among them, 35.89% of cases are due to coronary artery disease-induced regional wall motion abnormality (RWMA) and LV thrombus [3]. RWMA is significantly associated with undetermined stroke with embolic lesion-pattern (USELP). Reversibility of RWMA can be one of the most important predictors for the prevention of CE stroke [4].

## Case presentation

A 30-year-old female presented to the cardiology outpatient department with complaints of chest pain for one week, which was progressive and exertional in nature, consistent with Canadian Cardiovascular Society (CCS) Class IIIB angina. She had no comorbidities, no history of diabetes, hypertension, dyslipidaemia, or smoking, and no family history of premature coronary artery disease. There was no significant past medical or surgical history.

Examination

On general examination, she was alert, afebrile, and haemodynamically stable with blood pressure of 130/70 mmHg and a heart rate of 124 bpm. Cardiovascular examination revealed a normal first and second heart sound without added murmurs or gallops. Lungs were clear to auscultation, and there was no peripheral oedema or clinical evidence of systemic embolisation.

Investigations

Electrocardiogram (ECG)

**Figure 1:**
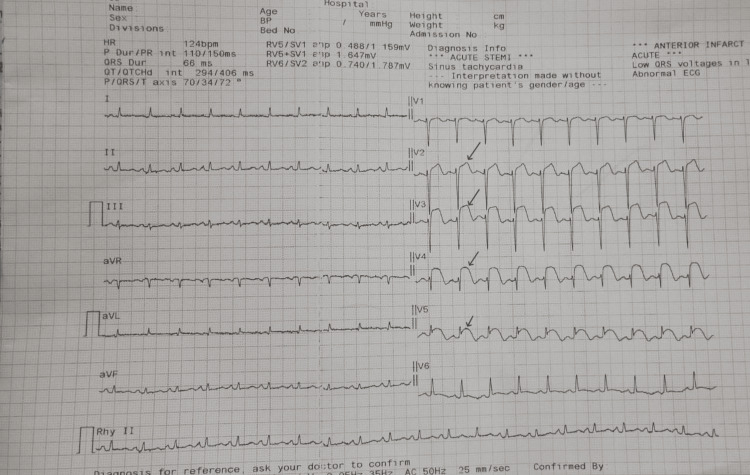
Baseline electrocardiogram. Twelve-lead ECG at presentation showing ST-segment elevation in the anterior leads, consistent with an acute anterior wall ST-elevation myocardial infarction.

Sinus rhythm with ST-segment elevation in the anterior precordial leads (V1-V4) (Figure 1), consistent with an acute anterior wall ST-elevation myocardial infarction (STEMI).

Cardiac Biomarkers

Elevated high-sensitivity troponin I levels confirm myocardial infarction.

Laboratory Profile

Complete blood count, renal function, electrolytes, and coagulation profile were within normal limits.

Echocardiography

**Figure 2:**
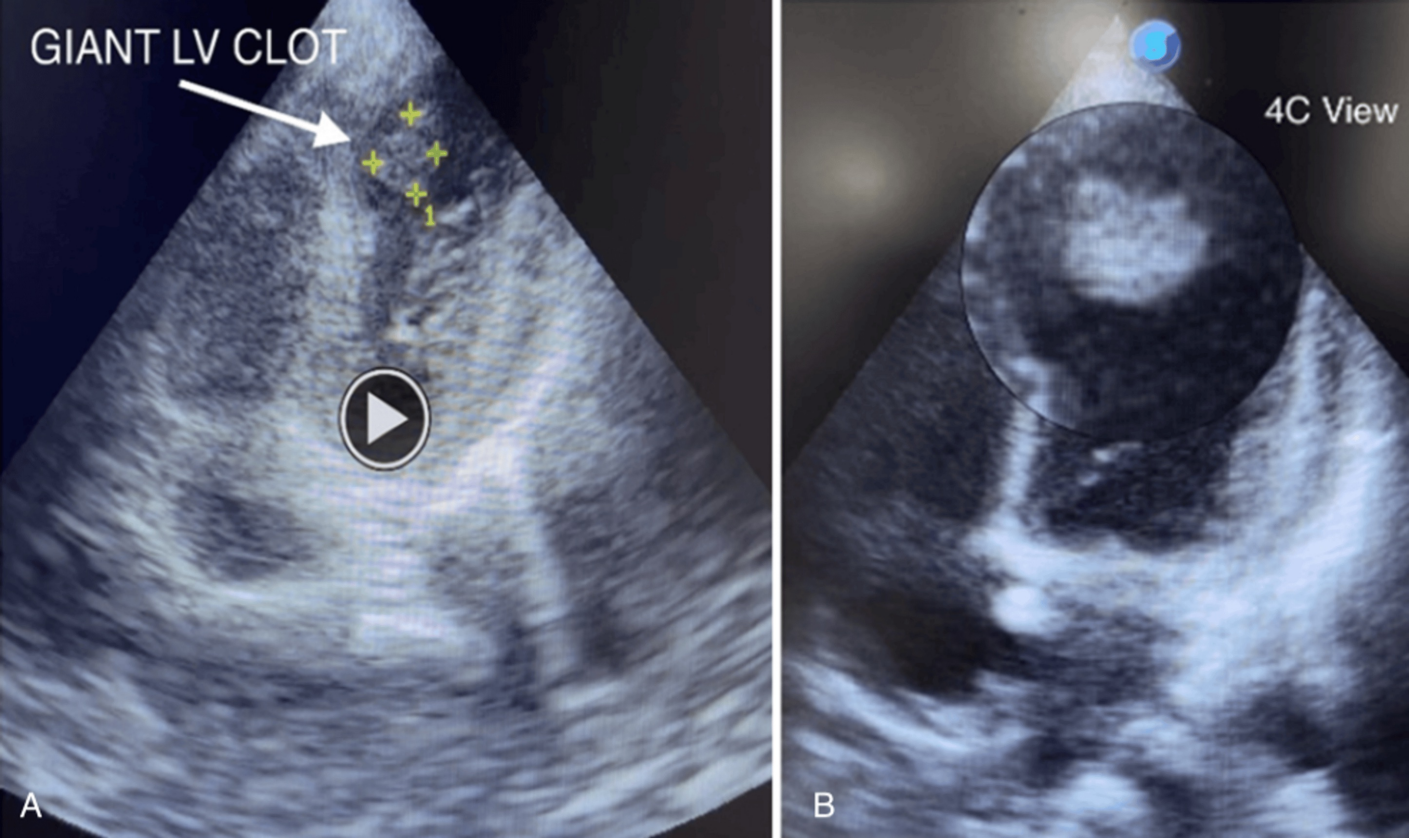
(A-B) Hypokinetic apical septal region with a large apical left ventricular thrombus (arrow).

2D transthoracic echocardiography revealed a severely hypokinetic septal-apical region with the presence of a large LV apical thrombus (Video 1 and Figure 2). Basal and mid-septal thickness were preserved, with overall left ventricular ejection fraction (LVEF) preserved at ~45%.

**Video 1:** Baseline echocardiography. Two-dimensional transthoracic echocardiography (apical four-chamber view) demonstrating a severely hypokinetic apical septal region with a large apical left ventricular thrombus.

Coronary Angiography

**Figure 3:**
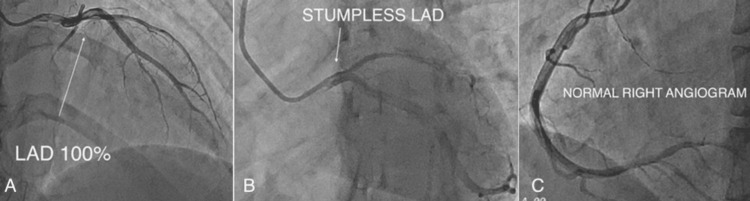
Coronary angiography (pre-PCI). Diagnostic coronary angiogram in the left anterior oblique view showing stumpless total occlusion of the proximal left anterior descending artery (LAD) (A), with normal left circumflex (LCx) (B) and right coronary arteries (RCA) (C). PCI: percutaneous coronary intervention

In view of ongoing angina and anterior STEMI, invasive coronary angiography was performed. It revealed a stumpless total occlusion of the proximal left anterior descending artery (LAD) (Figure 3A), while the left circumflex (LCx) (Figure 3B) and right coronary artery (RCA) (Figure 3C) were angiographically normal.

Given the ongoing angina, 100% proximal LAD occlusion, and high risk of further deterioration in LV function, the patient was taken up for PCI of the LAD.

Procedure

PCI to the LAD was performed via right radial access.

Vascular Access and Guide Engagement

A 6F radial sheath was inserted. A 6F Judkins left guiding catheter (Cordis, USA) was used to engage the left main coronary artery. Engagement was challenging due to poor visualisation of the proximal LAD stump.

Marker Wire Placement

**Figure 4:**
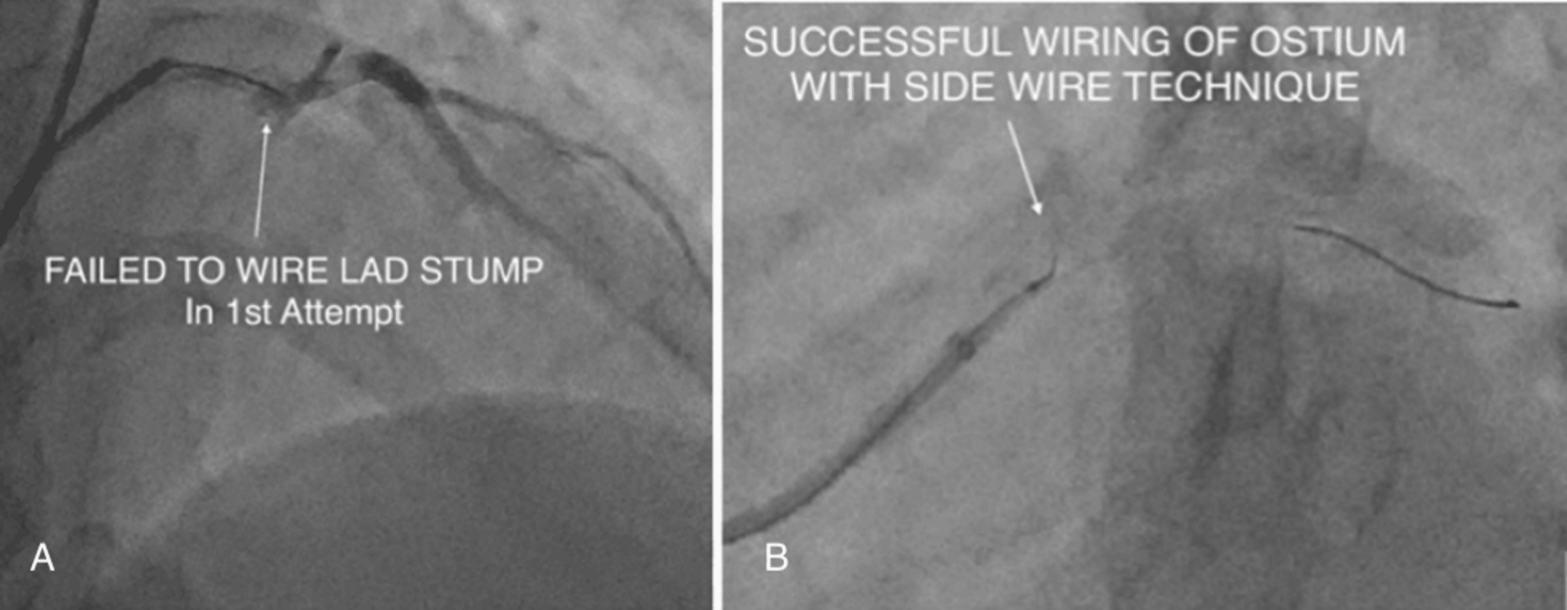
(A-B) Sion Blue guidewire positioned in the distal ramus branch as a marker. LAD: left anterior descending artery

A Sion Blue guidewire (Asahi Intecc, Japan) was first placed into the distal ramus branch to serve as a fluoroscopic marker and provide support (Figure 4).

Lesion Crossing

**Figure 5:**
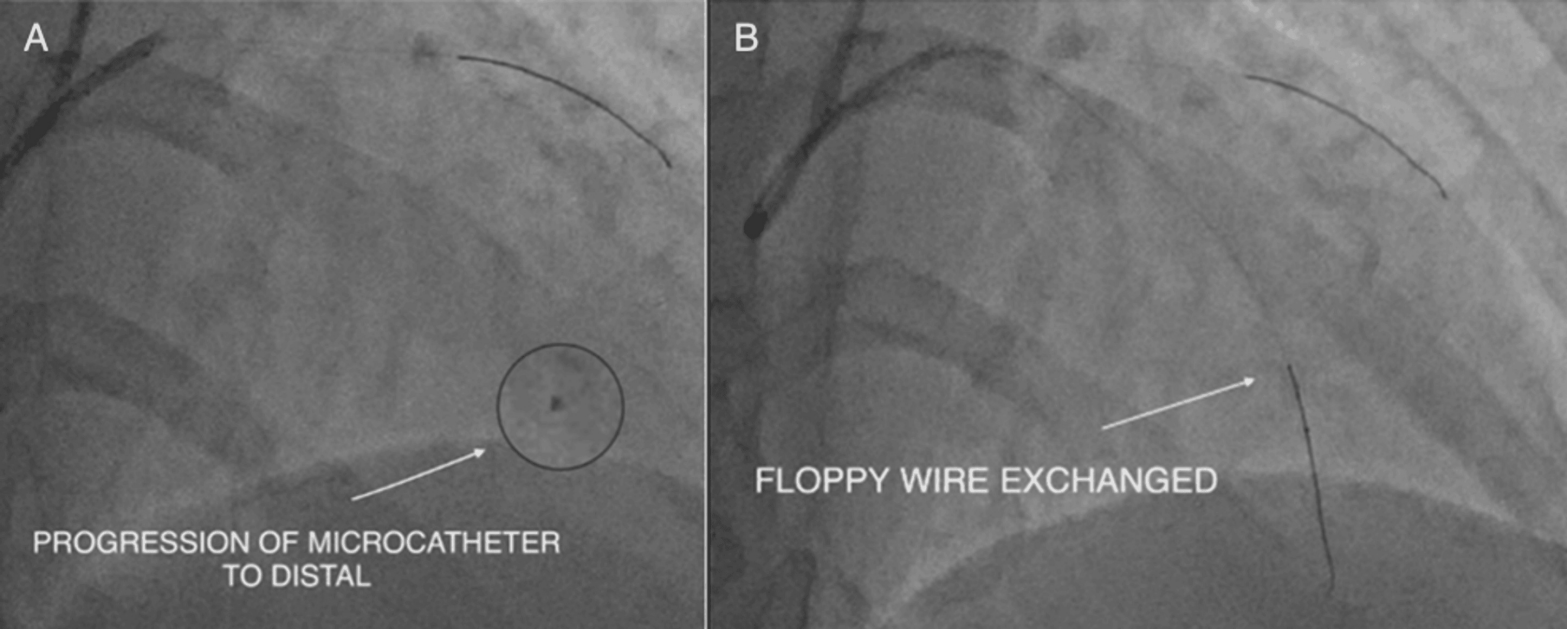
(A-B) Finecross microcatheter used to support guidewire passage across the LAD occlusion. LAD: left anterior descending artery

A Finecross microcatheter (Terumo, Japan) was advanced into the mid-left main over a floppy wire. A Fielder XT guidewire (Asahi Intecc, Japan) was manipulated through the occluded stumpless ostial LAD using dedicated wiring techniques (Figure 5A). After careful probing and contrast-assisted localisation of the true lumen, the wire successfully crossed the lesion and parked in the distal LAD.

Wire Exchange

The Finecross microcatheter was advanced into the distal LAD, and the Fielder XT wire was exchanged for a Sion Blue wire for enhanced support and device delivery (Figure 5B).

Lesion Preparation

**Figure 6:**
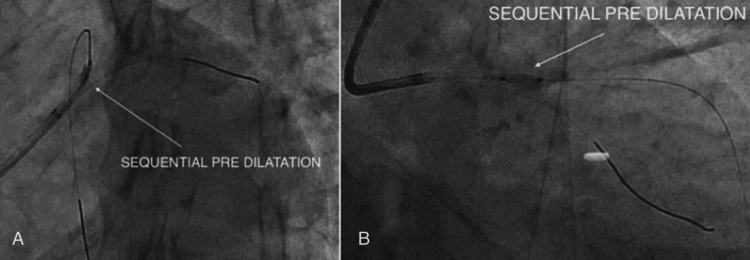
(A-B) Predilatation performed using Mini Trek balloons.

Sequential pre-dilatation of the ostio-proximal LAD lesion was performed with a 2.0 × 12 mm semi-compliant Mini Trek balloon (Abbott Vascular, USA) (Figure 6A), followed by a 2.5 × 15 mm semi-compliant Mini Trek balloon (Figure 6B) at nominal pressures. Adequate lesion expansion was achieved.

Stent Deployment

**Figure 7:**
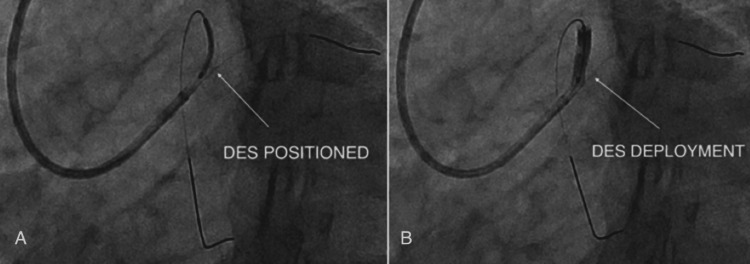
(A-B) Deployment of a 3.0 × 24 mm drug-eluting stent in the proximal LAD. LAD: left anterior descending artery

A 3.0 × 24 mm Xience drug-eluting stent (Abbott Vascular, USA) (Figure 7A) was positioned across the ostio-proximal LAD lesion and deployed successfully (Figure 7B).

Post-dilatation

**Figure 8:**
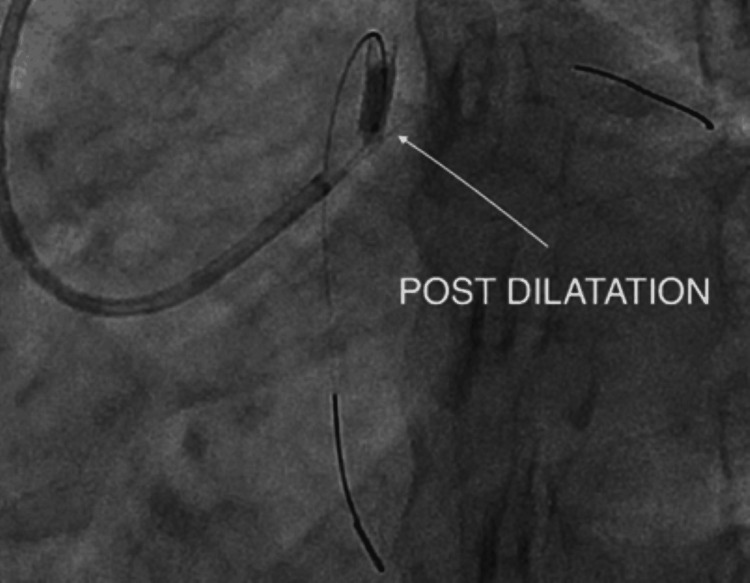
Post-dilatation using a 3.0 × 12 mm Mini Trek non-compliant balloon.

The stented segment was optimised using a 3.0 × 12 mm Mini Trek non-compliant balloon at high pressure to ensure optimal stent expansion and apposition (Figure 8).

Final Angiographic Result

**Figure 9:**
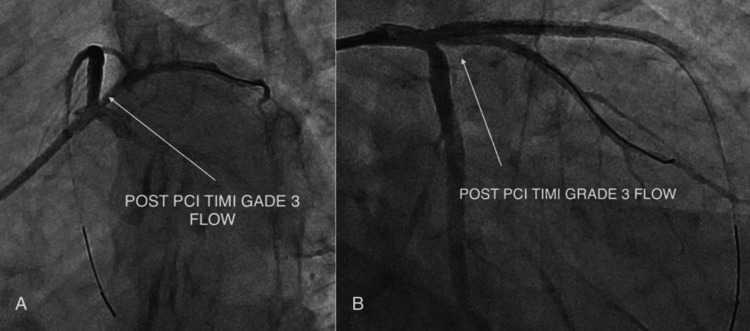
(A-B) Final angiogram demonstrating restored Thrombolysis in Myocardial Infarction (TIMI) grade III flow. PCI: percutaneous coronary intervention

Post-intervention angiography demonstrated a well-expanded stent with Thrombolysis in Myocardial Infarction (TIMI) grade III flow in the LAD and no residual stenosis or complications (Figure 9).

Peri-Procedural Course

The procedure was uneventful. No perforation, dissection, or side branch compromise occurred. The patient tolerated the procedure well. The patient was evaluated for all the possible causes of young CAD, but all tests were clear, and no significant findings were detected.

Post-procedure Management

In view of the large LV apical thrombus, the patient was initiated on triple antithrombotic therapy: aspirin, clopidogrel, and rivaroxaban. She was monitored in the coronary care unit and discharged after 48 hours of stable recovery, and a cardiac MRI (short-axis view) (Video 2) at two months demonstrated the absence of apical LV thrombus and improved regional contractility, confirming the resolution seen on echocardiography.

**Video 2:** Cardiac MRI (short-axis view) at two months demonstrating the absence of apical LV thrombus and no visible late gadolinium enhancement or regional motion wall abnormality. LV: left ventricular

## Discussion

This case highlights several important learning points.

Mechanism of thrombus formation

In this patient, apical hypokinesia due to proximal LAD occlusion led to regional stasis and thrombus formation. Similar mechanisms have been described in large anterior myocardial infarction [1,5]. In the setting of the abovementioned case, it followed the classical criteria of Virchow’s triad. Here, infarction led to hypoxic damage to the endocardium, hypokinesia led to vascular stasis, and lastly, the patient was in a hypercoagulable state due to increased prothrombotic factors like von Willebrand factor [6].

Role of PCI

By restoring the flow in the LAD, regional wall motion improved, reducing the vascular stasis and improving the endothelial dysfunction. Prior studies have shown that revascularisation reduces LV remodelling and thrombus risk [7]. A very early revascularisation procedure becomes the key determining factor for re-establishment of myocardial function and contractility [8]. RWMA and contractility improved significantly by the end of 30 days post-revascularisation in comparison to the baseline pre-procedure evaluation, and the wall motion score index also followed the same line of improvement [9].

Role of anticoagulation

The role of anticoagulation was the only mode of management of LV thrombus, but the effect of revascularisation on LV thrombus was not objectively explored. Continuation of anticoagulation therapy as the sole modality won't treat the cause of the inciting event, but can act as a prophylaxis. Even though non-vitamin K oral anticoagulants (NOACs) are routinely used in clinical practice, there are limited data comparing the efficacy of a NOAC versus a vitamin K antagonist (VKA) in the setting of LV thrombus [10]. In the above-mentioned case of post-management of acute STEMI, anticoagulation was started with NOACs such as rivaroxaban [9,11,12]. When compared to conventional VKAs, these drugs are more efficacious and have better safety profiles, lowering the risk of intracranial bleeding, and don't require vigorous monitoring.

Unique outcome

The combination of successful PCI and NOAC therapy in this young patient resulted in complete thrombus resolution and improvement in overall contractility and reversibility of cardiac function within two months, which was confirmed by both echocardiography and cardiac MRI.

## Conclusions

Early revascularisation of infarct-related LAD combined with NOAC therapy may promote complete resolution of LV thrombus, even in cases of large apical involvement, and prevent devastating embolic complications in young patients. Severe apical hypokinesia in anterior STEMI may predispose to LV thrombus formation. Early LAD revascularisation improves regional wall motion and reduces thrombus persistence. NOACs, in combination with PCI, can be highly effective in LV thrombus management. Comprehensive imaging follow-up is valuable for confirming thrombus resolution.

In our patient, one timely intervention led to the prevention of two major life-threatening events: the progression of LV dysfunction and early decreased overall cardiac contractility and progression to ischaemic cardiomyopathy and embolic ischaemic stroke, which in turn would have led to permanent neurological deficits.
